# Targets and mechanisms of epigenetic regulation in the temperate cereal vernalisation process

**DOI:** 10.3389/fpls.2025.1520593

**Published:** 2025-08-05

**Authors:** Tomas Daneels, Gustavo Martinez-Barrales, Cederic Bosmans, Koen Geuten

**Affiliations:** Department of Biology, KU Leuven, Leuven, Belgium

**Keywords:** vernalisation, temperate cereals, histone modification, VRN1, devernalisation

## Abstract

Vernalisation is a prolonged cold exposure that synchronises flowering with favourable seasonal conditions, protecting reproductive development from winter stress and optimising crop yields. By ‘recording’ winter, this biological process displays memory and becomes progressively more difficult to reverse. In the well-studied model Arabidopsis, vernalisation epigenetically silences the gene locus of the floral repressor *FLC*. Temperate cereals, including crops such as wheat and barley, respond to a similar vernalisation cue with a memory property and recent functional studies also support an epigenetic mechanism. Current evidence points to the flowering promoter *VRN1* as the primary site for storing this memory. Because vernalisation in cereals appears to rely on epigenetic activation of *VRN1*, rather than repression as in Arabidopsis, the specific histone marks responsible for storing this epigenetic memory are possibly different. This highlights the need for further research to identify the specific genes and histone modifications involved, and to fully elucidate the mechanisms underlying vernalisation memory in cereals. The goal of this review is to synthesise recent advances in our understanding of the epigenetic regulation of vernalisation in temperate cereals. Therefore, this review focuses on the roles of key genes such as *VRN1*, *VRN3*, and *ODDSOC2*, and examines the dynamic chromatin landscape associated with vernalisation-induced flowering. In particular, we investigate the possible interplay of chromatin marks involved in the epigenetic activation of *VRN1*. By synthesising current knowledge and highlighting unresolved questions, this review aims to provide a framework for future research in the field of cereal vernalisation memory.

## Introduction

Plants, as immobile organisms, have evolved sophisticated mechanisms to sense and integrate environmental cues, ensuring that key developmental transitions occur under favourable conditions. A prime example of such a sophisticated mechanisms is vernalisation, the requirement for a prolonged period of cold to induce flowering (Chouard, 1960; Sung and Amasino, 2004). By gradually sensing prolonged exposure to cold temperatures, plants initiate physiological changes that accelerate the transition from the vegetative to the reproductive stage, ensuring that flowering occurs only in spring. This adaptive strategy helps to protect developing floral structures from harsh winter conditions and prevents premature seed set (Sung and Amasino, 2004; Trevaskis et al., 2007). Vernalisation is particularly critical for winter varieties of temperate cereal crops, such as wheat, barley, and oats, that are cultivated in regions with distinct seasonal changes and temperate climates. These winter varieties are preferred in these climates because they demonstrate superior yield, explained by the prolonged vegetative growth they undergo before transitioning to reproduction. The ability to synchronise flowering with favourable seasonal conditions not only underscores the fundamental biological importance of vernalisation but also highlights its critical role in agriculture, where flowering time directly influences crop yield and stability (Wang et al., 2023; Wu et al., 2017). As climate patterns grow increasingly unpredictable, understanding the mechanisms of vernalisation acquires further importance in developing resilient crop varieties.

Through the mechanism behind vernalisation, plants acquire the ability to “remember” winter. Because epigenetic mechanisms change at a slower rate than e.g. transcriptional changes, the memory property of vernalisation is probably encoded at the epigenetic level in the different species that developed a vernalisation response. Vernalisation research in *Arabidopsis thaliana* (Arabidopsis) has been pivotal in uncovering the molecular basis of this epigenetic memory. In this species, the floral repressor *FLOWERING LOCUS C* (*FLC*) is epigenetically silenced during extended cold, thereby lifting repression and permitting flowering when temperatures rise (Song et al., 2012; Sung and Amasino, 2004). The degree of histone modification at the *FLC* locus quantitatively reflects the duration of cold exposure, acting as a molecular record that ensures flowering only after the vernalisation requirement is met. This model system has also illuminated the roles of specific histone modifiers, non-coding RNAs, and feedback mechanisms that stabilise *FLC* repression and maintain the vernalised state (Whittaker and Dean, 2017).

In contrast, the mechanisms underlying vernalisation memory in temperate cereals differ in two fundamental ways. First, while Arabidopsis stores vernalisation memory through the stable deposition of repressive histone marks at a single locus, it remains unclear whether vernalisation memory in cereals is confined to *VRN1* or involves coordinated regulation across multiple genes and genomic loci. Second, rather than repressing a floral inhibitor, temperate cereals appear to rely on the epigenetic activation of a floral promoter gene, *VERNALISATION1* (*VRN1*), which promotes the transition to flowering (Dixon et al., 2019; Shitsukawa et al., 2007; Trevaskis et al., 2003; Yan et al., 2003). Furthermore, the specific histone modifications responsible for maintaining this irreversible active state in cereals have yet to be identified. These fundamental differences underscore the complexity and diversity of vernalisation mechanisms among plant species and highlights significant gaps in our current understanding of cereal vernalisation memory.

In this review, we synthesise recent advances in the field, with a particular emphasis on the roles of key genes such as *VRN1*, *VRN3*, and *ODDSOC2*, and the dynamic chromatin landscape that underpins vernalisation-induced flowering. We discuss the interplay of chromatin marks involved in the epigenetic activation of *VRN1*, highlight unresolved questions, and propose a framework for future research to advance our understanding of vernalisation memory in cereals.

## Genetic basis of vernalisation

The transition to flowering in temperate cereals is a tightly regulated developmental process that ensures reproductive development takes place only when environmental conditions are optimal. This precise timing is orchestrated by a complex genetic network centred around the *VERNALISATION* (*VRN*) genes: *VRN1*, *VRN2*, and *VRN3* (Dennis and Peacock, 2009; Greenup et al., 2009). Notably, these genes are distinct from the vernalisation genes of Arabidopsis, even though their names are identical. The fact that different genes control the same process suggests that the vernalisation pathways in temperate cereals and dicots have evolved independently (Andrés and Coupland, 2012; Dennis and Peacock, 2009).

**Figure 1:**
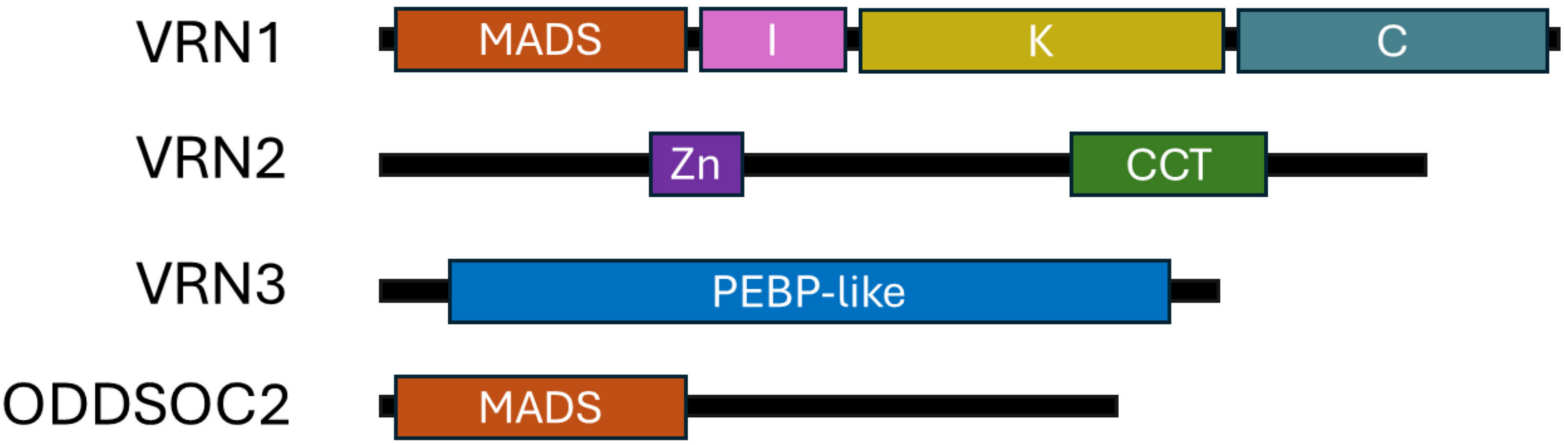
Structural domain composition *VRN1*, *VRN2*, *VRN3*, and *ODDSOC2*. Schematic representation of the structural domains present in *VRN1*, *VRN2*, *VRN3*, and *ODDSOC2*, showing the arrangement of MADS, intervening (I), keratin-like (K), C-terminal, and PEBP domains to highlight differences in domain composition among these key flowering regulators from Brachypodium distachyon. Created in BioRender. Daneels, T. (2025) https://BioRender.com/lo4b3yy.

**Figure 2:**
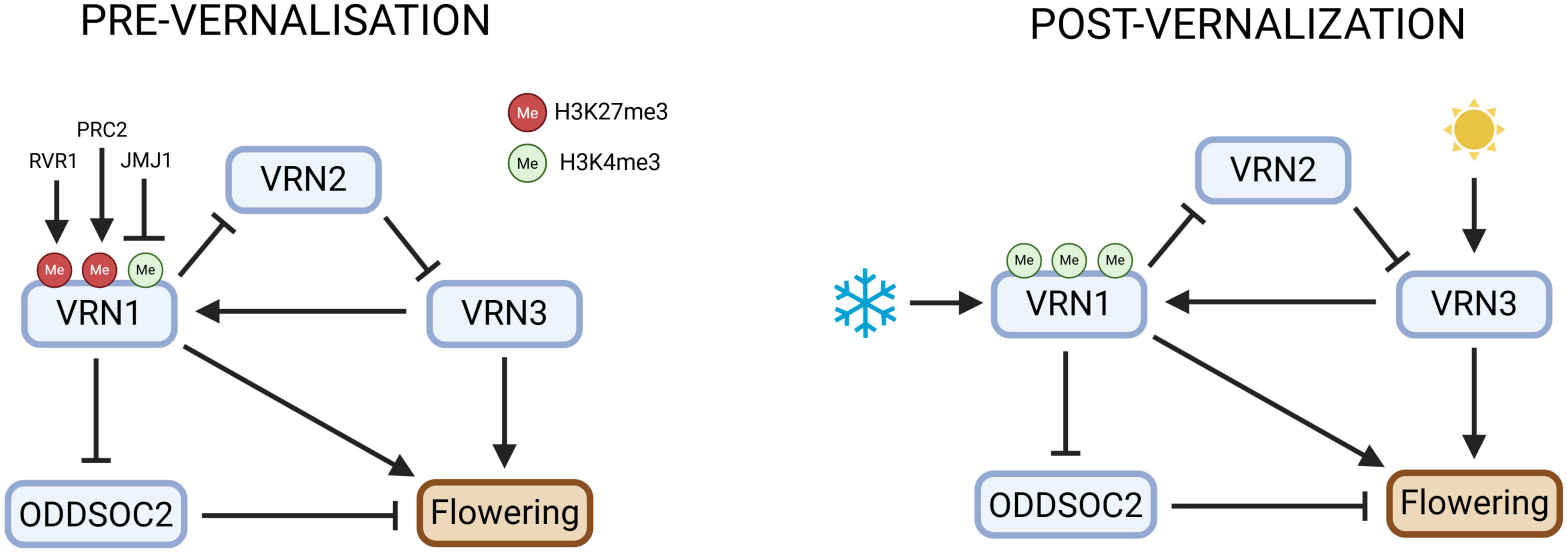
Genetic and epigenetic network controlling vernalisation and flowering in temperate cereals. This figure illustrates the genetic and epigenetic regulation of flowering in temperate cereals in response to cold and light: before vernalisation, *VRN1* is repressed by high H3K27me3 (via PRC2/EZL1), as well as by the pre-vernalisation repressors *RVR1* and *JMJ1*, with JMJ1 demethylating H3K4 to further silence *VRN1*; this maintains active *VRN2* and *ODDSOC2*, which keep *VRN3* inhibited. During and after cold exposure, *VRN1* is activated through loss of H3K27me3 and gain of H3K4me3, leading to stable expression and winter memory; *VRN1* then represses *VRN2* and *ODDSOC2*, releasing *VRN3* from inhibition. *VRN3* activation is marked by increased H3K4me3, especially under long days. *ODDSOC2* repression involves increased H3K27me3 in Brachypodium, while in wheat and barley, its downregulation is associated with reduced H3K4me3 and is mediated by *VRN1*. These coordinated genetic and chromatin changes, regulated by *EZL1*, *RVR1*, and *JMJ1*, integrate cold and photoperiod signals to precisely control flowering time. Created in BioRender. Daneels, T. (2025) https://BioRender.com/0z70j0s.

*VRN1* encodes a protein from the MADS-box transcription factor family, homologous to the Arabidopsis genes *AP1* and *FUL* (Trevaskis et al., 2003; Yan et al., 2003). *VRN1* possesses a characteristic MIKC-type domain structure, which includes four conserved domains: the MADS (M), Intervening (I), Keratin-like (K), and C-terminal (C) domains (**Figure 1**) (Egea-Cortines et al., 1999; Melzer et al., 2009; Riechmann and Meyerowitz, 1997; Shore and Sharrocks, 1995). The MADS domain is a highly conserved DNA-binding motif crucial for recognising and regulating the expression of target genes involved in floral development (West et al., 1997). The I domain provides specificity for protein dimerisation, helping *VRN1* select its interaction partners. The K domain is essential for protein-protein interactions, facilitating the formation of higher-order complexes with other MADS-box proteins, which is vital for the regulation of flowering. The C-terminal domain contributes to transcriptional activation and further protein-protein interactions. Importantly, MADS-box transcription factors such as *VRN1* often function not as single proteins but as part of multimeric complexes, typically dimers or higher-order assemblies, which enable combinatorial control of gene expression and allow for precise regulation of developmental processes (Immink et al., 2009; Smaczniak et al., 2012; Theißen and Saedler, 2001). In cereals, *VRN1* directly binds to promoters of key flowering genes, including *VRN3*, and flowering repressors such as *VRN2* and *ODDSOC2*, thereby promoting flowering after vernalisation (**Figure 2**) (Chen and Dubcovsky, 2012; Deng et al., 2015; Oliver et al., 2009; Woods et al., 2016). This modular MIKC structure enables *VRN1* to function as a transcriptional regulator, integrating vernalisation signals by altering gene expression profiles that trigger the transition from vegetative to reproductive growth (Distelfeld and Dubcovsky, 2009; Trevaskis et al., 2007).

*VRN2*, in contrast, encodes a CCT domain protein, containing both a zinc finger and a CCT (*CONSTANS*, *CONSTANS-like*, and *TIMING OF CAB1*) domain (**Figure 1**) (Fu et al., 2005; Yan et al., 2006). The CCT domain is essential for nuclear localisation and protein-protein interactions, particularly with NF-Y transcription factors, and is a hallmark of proteins involved in photoperiod and circadian regulation (Griffiths et al., 2003; Tiwari et al., 2010). The repressor activity of *VRN2* depends on the integrity of the CCT domain, and mutations in this region can abolish the vernalisation requirement (Chen and Dubcovsky, 2012; Fu et al., 2005). The zinc finger domain contributes to DNA binding, further supporting *VRN2’s* function as a floral repressor.

*VRN3* encodes a member of the phosphatidylethanolamine-binding protein (PEBP) family, homologous to Arabidopsis *FLOWERING LOCUS T* (**Figure 1**) (Yan et al., 2006). The PEBP domain enables *VRN3* to act as a mobile florigen, interacting with 14-3–3 proteins and the bZIP transcription factor FD to form a complex that migrates from leaves to the shoot apical meristem, where it activates downstream flowering genes, including *VRN1* (Distelfeld and Dubcovsky, 2009; Yan et al., 2006).

*ODDSOC2*, along with *ODDSOC1* and *MADS37*, represents a group of *FLC-like* MADS-box transcription factors in cereals. While *ODDSOC2* is a truncated MADS-box protein, it retains the core DNA-binding domain necessary for its function as a potent floral repressor (**Figure 1**) (Greenup et al., 2010).

The structural features of these proteins directly inform their genetic interactions, creating a tightly regulated feedback loop that integrates environmental cues to control flowering time (**Figure 2**). In autumn, *VRN2* is highly expressed and acts as a floral repressor by downregulating *VRN3*, thereby preventing premature flowering (Dubcovsky, 2005; Yan et al., 2004). In winter varieties of wheat and barley, *VRN1* is expressed at low levels prior to winter but is strongly induced by prolonged cold exposure, a response that is central to the vernalisation process (Dixon et al., 2019; Shitsukawa et al., 2007; Yan et al., 2003). This cold-induced upregulation of *VRN1* is essential for initiating the transition from vegetative to reproductive growth, and its high expression is maintained after vernalisation, ensuring that flowering is irreversibly initiated, after the plant has experienced sufficient winter cold. In contrast, spring varieties often carry deletions or mutations in the *VRN1* promoter or first intron, which allows *VRN1* to be expressed even in the absence of cold exposure, thereby reducing or abolishing the requirement for vernalisation (Fu et al., 2005; Muterko et al., 2016; Xu and Chong, 2018). The epigenetic regulation of *VRN1* is reminiscent of the *FLC* pathway in Arabidopsis: *VRN1* upregulation is associated with a decrease in repressive H3K27me3 (trimethylation of histone H3 at lysine 27) chromatin marks and an increase in active H3K4me3 (trimethylation of histone H3 at lysine 4) marks, supporting the importance of chromatin state changes in vernalisation-induced gene expression (Huan et al., 2018; Liu et al., 2023; Oliver et al., 2009). High levels of *VRN1* suppress *VRN2* expression, allowing *VRN3* activation and flowering. Notably, this *VRN1*-mediated suppression of *VRN2* is unique to wheat and barley, as in other pooid grasses such as Brachypodium, *VRN2* is not repressed by *VRN1* during or after vernalisation (Greenup et al., 2009; Woods et al., 2016).

With *VRN2* repressed, *VRN3* is upregulated, especially under long-day conditions in spring, a process further promoted by photoperiod genes such as PPD1 and CO (Chen and Dubcovsky, 2012; Dubcovsky et al., 2006). *VRN3*, acting as a mobile florigen, migrates to the shoot apex and directly promotes *VRN1* transcription, reinforcing its own activation through a positive feedback loop (Trevaskis et al., 2007; Turner et al., 2005; Yan et al., 2006). This feedback ensures a robust and irreversible commitment to flowering once vernalisation and favourable photoperiod conditions are met (A. Chen and Dubcovsky, 2012; Distelfeld and Dubcovsky, 2009).

*ODDSOC2*, as a floral repressor, is also tightly regulated by this network. Following vernalisation, *VRN1* is required to maintain the repression of *ODDSOC2*, ensuring that this repressor remains downregulated and thus permitting rapid flowering (Deng et al., 2015; Dixon et al., 2019; Greenup et al., 2010). In *vrn1* mutants or in cases of incomplete vernalisation, *ODDSOC2* expression can rise again, leading to delayed flowering and reduced plant growth. Mechanistically, *ODDSOC2* represses *FPF1-like genes*, which are positive regulators of floral development and cell elongation, providing a direct link between *ODDSOC2* activity and the inhibition of flowering (Greenup et al., 2010).

## Vernalisation memory

A classic example of vernalisation memory is found in Arabidopsis, where prolonged cold leads to the stable silencing of the floral repressor *FLC* (Bastow et al., 2004; Michaels and Amasino, 1999; Sheldon et al., 2000). During vernalisation, specific histone modifications accumulate at the *FLC* locus. Most notably, the repressive mark H3K27me3 spreads across the gene, locking *FLC* into a silent state that persists even after the plant returns to warmer temperatures (Angel et al., 2011; Bastow et al., 2004; De Lucia et al., 2008).

Histone methylation, such as H3K27me3, is a particularly stable modification. It can persist through cell divisions, providing the molecular basis for long-term epigenetic memory and ensuring continued repression of *FLC* after vernalisation (Cheung and Lau, 2005; Whittaker and Dean, 2017). In contrast, histone acetylation is highly dynamic and reversible, allowing for rapid but short-lived changes in gene activity (Chen and Tian, 2007). In addition, activating marks like H3K4me3 are reduced at *FLC* during vernalisation (De Lucia et al., 2008). Together, these histone modifications influence how tightly DNA is packaged around histone proteins, ultimately determining whether genes are accessible for transcription or kept in a silenced state.

Turning to temperate cereals, recent genome-wide studies have shown that vernalisation leads to widespread changes in histone modifications and gene expression at several important loci, including *VRN1*, *VRN3*, and *ODDSOC2* (Oliver et al., 2009; Sharma et al., 2017). While *VRN1* remains a central focus, these findings raise the possibility that the epigenetic memory of vernalisation in cereals could involve a broader network of genes, rather than being limited to a single locus. This highlights the need for further investigation in this area. Given this complexity, it is important to set out clear criteria to help identify which genes may be involved in storing vernalisation memory. In the next section, we outline such criteria, providing a framework for pinpointing candidate memory genes and guiding future research into the molecular basis of vernalisation memory in temperate cereals.

## Criteria for establishing epigenetic regulation

First, there should be phenotypic evidence from knockout lines that demonstrates the gene’s importance in the vernalisation process. In Arabidopsis, for instance, *FLC* knockout plants lose their vernalisation requirement and flower early, highlighting the gene’s pivotal role (Michaels and Amasino, 1999). Similar phenotypic evidence should be sought in cereals, where loss-of-function mutants in candidate genes would be expected to eliminate the vernalisation response. Second, there should be direct evidence of epigenetic changes at this gene locus during vernalisation. At *FLC*, vernalisation leads to the accumulation of repressive histone marks at the gene locus, which correlates with stable gene silencing (Bastow et al., 2004; Shindo et al., 2006; Sung and Amasino, 2005). Detecting comparable chromatin changes at candidate loci in cereals is crucial. Third, phenotypes in mutants of epigenetic regulators, such as histone modification writers and erasers, should be considered. In Arabidopsis, mutations in chromatin-modifying proteins that affect *FLC* silencing also disrupt vernalisation memory (Mylne et al., 2004). The identification of similar regulator mutants in cereals strengthens the case for the role of the corresponding epigenetic modifications in establishing memory. Fourth, there should ideally be evidence of physical interaction between the regulators and the gene locus. For *FLC*, direct binding of chromatin-modifying proteins to the gene during vernalisation has been demonstrated (Angel et al., 2011; Franco-Echevarría et al., 2023; Schulten et al., 2024). Evidence of such interactions at cereal loci would further support their involvement. These criteria, illustrated by the case of *FLC* in Arabidopsis, provide a robust framework for dissecting the molecular basis of vernalisation memory in cereals.

## Targets of epigenetic regulation

Having outlined the criteria for identifying genes that may store the epigenetic memory of vernalisation, we now evaluate the main candidate genes in temperate cereals. Genome-wide studies have shown that vernalisation triggers widespread changes in histone modifications and gene expression (Oliver et al., 2009). However, while many genes display such changes during cold treatment, only a few have been directly linked to a vernalisation phenotype. Applying our criteria helps distinguish which genes are already known to be central to the epigenetic regulation of flowering by vernalisation. Currently, the strongest candidates for storing vernalisation memory in temperate cereals are *VRN1*, *VRN3*, and *ODDSOC2* (**Table 1**).

**Table 1:** Criteria for assessing epigenetic regulation of genes in vernalisation.

Criterion	Description	VRN1	VRN3	ODDSOC2
Vernalisation-related phenotype	The gene exhibits a mutant phenotype that fully or partially affects the vernalisation response (e.g., altered flowering time).	✓ᯅ	✓ᯅ	✓ᯅ
Epigenetic modification	Epigenetic changes (e.g., histone modifications, DNA methylation) are observed at the gene locus during or after vernalisation.	✓ᯅ	✓ᯅ	✓ᯅ
Regulator-dependent epigenetic phenotype	A mutant in an epigenetic regulator results in altered epigenetic status and/or expression of the gene, affecting vernalisation.	✓ᯅ	✗ᯅ	✗ᯅ
Physical interaction with the regulator	The epigenetic regulator physically associates with the gene locus (e.g., via ChIP, DAP-seq), confirming direct regulation.	✓ᯅ	✗ᯅ	✗ᯅ

Summary of the four key criteria used to evaluate whether a gene is epigenetically regulated during the vernalisation process, including phenotypic effects, epigenetic modifications, regulatory dependencies, and physical interactions with epigenetic regulators.

Knockout lines of *VRN1* show a pronounced delay in flowering, confirming its essential role in the vernalisation response (Trevaskis et al., 2003; Yan et al., 2003). Epigenetic regulation of *VRN1* during vernalisation is characterised by dynamic changes in histone marks, which are orchestrated by specific chromatin modifiers. Before vernalisation, the *VRN1* locus in Brachypodium, barley, and wheat is marked by high levels of the repressive histone modification H3K27me3, which keeps the gene silent (see **Table 2** for a detailed overview of epigenetic changes at the *VRN1* locus) (Lomax et al., 2018; Oliver et al., 2009). During prolonged cold exposure, these repressive marks are progressively reduced, while activating marks such as H3K4me3, H3K36me3 (trimethylation of histone H3 at lysine 36), and H3K27Ac (acetylation of histone H3 at lysine 27) become increasingly enriched at the *VRN1* promoter and first exon. The extent of H3K27me3 loss and H3K4me3 gain is closely linked to the duration of cold, correlating with a cumulative and possibly stable activation of *VRN1*.

**Table 2:** Epigenetic modifications at the *VRN1* locus during vernalisation in Brachypodium, barley, and wheat.

Species	Accession	Modification	Observation during vernalisation	Citation
Brachypodium	ABR1	H3K4me3	Upregulated	(Huan et al., 2018)
	Bd21-3	H3K4me3	Upregulated	(Mayer and Charron, 2021)
	Bd21-3	H3K4me2	Downregulated	(Liu B. et al., 2024)
	Bd21-3	H3K4me3	Upregulated	(Liu B. et al., 2024)
	ABR1	H3K27me3	Downregulated	(Huan et al., 2018)
	Bd21-3	H3K27me3	Downregulated	(Lomax et al., 2018)
	Bd21-3	H3K27me3	Downregulated	(Mayer and Charron, 2021)
	Bd21-3	H3K27me3	Downregulated	(Woods et al., 2017)
Barley	Sonja	H3K4me3	Upregulated	(Oliver et al., 2009)
	Sonja	H3K27me3	Upregulated	(Oliver et al., 2009)
Wheat	AK58	H3K27Ac	Upregulated	(Liu Y. et al., 2024)
	Cv Norstar	H3K4me3	Upregulated	(Diallo et al., 2012)
	Cv Manitou	H3K4me3	Downregulated	(Diallo et al., 2012)
	Fielder/KN199	H3K4me3	Upregulated	(Li et al., 2024)
	AK58	H3K4me3	Upregulated	(Liu Y. et al., 2024)
	JH9	H3K4me3	Upregulated	(Xiao et al., 2014)
	Cultivar 2174	H3K4me3	No changes	(Xie et al., 2021)
	KN9204	H3K36me3	Upregulated	(Liu et al., 2023)
	AK58	H3K36me3	Upregulated	(Liu Y. et al., 2024)
	Cv Norstar/Cv Manitou	H3K27me3	No changes	(Diallo et al., 2012)
	Cultivar 2174	H3K27me3	Downregulated	(Xie et al., 2021)
	KN9204	H3K27me3	Downregulated	(Liu et al., 2023)
	AK58	H3K27me3	Downregulated	(Liu Y. et al., 2024)

This table summarises the four best-described histone modifications during vernalisation at the *VRN1* locus, three of which activate transcription (H3K4me3, H3K27Ac, and H3K36me3) and one that suppresses transcription (H3K27me3). All accessions are winter accessions, except from the wheat spring accession Fielder.

The establishment and maintenance of histone marks is mediated by several key epigenetic regulators. *ENHANCER OF ZESTE-LIKE 1* (*EZL1*), a homolog of CURLY LEAF in Arabidopsis, acts as the catalytic subunit of the Polycomb Repressive Complex 2 (PRC2), which deposits H3K27me3 at the *VRN1* locus (Lomax et al., 2018). In Brachypodium, mutations in *EZL1* result in reduced H3K27me3 levels and early flowering even without vernalisation, highlighting its role in repressing *VRN1* prior to cold exposure (Lomax et al., 2018). However, since PRC2 does not deposit activating marks, EZL1 is mainly involved in establishing the vernalisation requirement rather than maintaining post-vernalisation memory.

*REPRESSOR OF VERNALISATION1* (*RVR1*) is another key regulator that helps maintain H3K27me3 levels at the *VRN1* locus before vernalisation in Brachypodium (Woods et al., 2017). Loss-of-function mutations in RVR1 lead to an overall reduction in H3K27me3, which mimics the vernalised state and results in early flowering even without cold exposure (Woods et al., 2017). This demonstrates that RVR1 acts as a pre-vernalisation repressor, ensuring that *VRN1* is only activated after sufficient cold exposure. Its inactivation during vernalisation permits the epigenetic activation of *VRN1*, thereby linking environmental cues to reproductive timing (Woods et al., 2017).

JUMONJI1 (JMJ1) is a Jumonji C (JmjC)-domain-containing histone demethylase that plays a crucial role in regulating flowering time in Brachypodium (Liu B. et al., 2024). This enzyme specifically removes methyl groups from the histone marks H3K4me2 and H3K4me3 at target genes, including the key flowering regulator *VRN1* (Liu B. et al., 2024). JMJ1 helps to repress *VRN1* before vernalisation by maintaining low levels of activating histone marks at the *VRN1* locus. When JMJ1 is mutated or absent, H3K4me2 and H3K4me3 accumulate at *VRN1*, leading to increased *VRN1* expression and earlier flowering (Liu B. et al., 2024). Chromatin immunoprecipitation experiments have shown that JMJ1 directly binds to the chromatin of *VRN1* and another flowering gene, *INDETERMINATE1 (ID1)*, confirming its direct regulatory role (Liu B. et al., 2024). Genome-wide analyses indicate that H3K4me3 is generally associated with higher gene expression, while H3K4me2 is negatively correlated with transcript levels in Brachypodium (Liu B. et al., 2024). Thus, JMJ1-mediated demethylation fine-tunes the chromatin environment at *VRN1*, ensuring that flowering occurs at the appropriate time in response to seasonal cues (Liu B. et al., 2024).

*VRN1* fulfills the criteria for an epigenetic memory locus in vernalisation. Numerous studies have shown that *VRN1* remains stably upregulated following vernalisation, supporting its role in the long-term memory of winter conditions (Lomax et al., 2018; Woods et al., 2017). In some cases, *VRN1* expression may decrease slightly when plants return to warm temperatures, but this typically occurs only after insufficient cold exposure, resulting in incomplete vernalisation and potential loss of memory (Woods et al., 2017).

The regulation of *VRN1* expression involves a tight interplay between repressive and activating histone marks, coordinated by the epigenetic modifiers EZL1, RVR1, and JMJ1. EZL1 and RVR1 maintain repression of *VRN1* through H3K27me3 prior to vernalisation, while JMJ1 reinforces this silent state by removing activating histone marks (Lomax et al., 2018; Woods et al., 2017). During cold exposure, the coordinated reduction of repressive marks and accumulation of activating marks at *VRN1* enable stable gene activation (Lomax et al., 2018; Woods et al., 2017). Mutations in these histone-modifying regulators alter flowering time, and direct interactions between these complexes and the *VRN1* locus have been demonstrated. However, although these mutants clearly disrupt histone modification patterns and flowering time, they do not yet fully explain how the stable epigenetic memory of vernalisation is established and maintained at the *VRN1* locus. The precise molecular mechanisms underlying the long-term maintenance of this memory remain unresolved and require further investigation.

*VRN3* is another important gene associated with the vernalisation response and is considered a potential target for epigenetic regulation. While knockout lines for *VRN3* have not yet been generated, Yan et al. (2006) demonstrated that overexpression of *VRN3* in wheat results in early flowering. However, the evidence for epigenetic regulation of *VRN3* varies across species. While a single study in barley did not observe changes in epigenetic marks (Oliver et al., 2009), evidence for epigenetic regulation of *VRN3* has been reported in other species, such as wheat and Brachypodium (Huan et al., 2018; Liu Y. et al., 2024). Notably, in Brachypodium, these epigenetic changes were observed only when vernalisation was combined with inductive long-day conditions, making it unclear whether vernalisation alone is sufficient to induce such regulation (Huan et al., 2018). As a result, while *VRN3* can be epigenetically regulated in some temperate cereals, it does not fully meet all four proposed criteria.

*ODDSOC2* acts as a flowering repressor that is downregulated during vernalisation. In Brachypodium, its stable repression is associated with increased H3K27me3 at the *ODDSOC2* locus (Sharma et al., 2017). In contrast, in barley and wheat, *ODDSOC2* downregulation during vernalisation does not involve direct H3K27me3-mediated silencing. Instead, in barley, repression is primarily achieved through *VRN1*-mediated transcriptional regulation, with additional evidence for epigenetic involvement as vernalisation reduces H3K4me3 levels at the *ODDSOC2* locus, correlating with decreased expression (Deng et al., 2015; Greenup et al., 2010). In wheat, the initial downregulation of *ODDSOC2* is triggered by cold exposure independently of *VRN1*, but long-term repression after vernalisation depends on *VRN1* activity, again without evidence for H3K27me3 involvement (Chen and Dubcovsky, 2012; Greenup et al., 2010; Liu et al., 2023).

To summarise the evaluation of these candidates, *VRN1* demonstrates the strongest evidence for correlative epigenetic modifications alongside stable changes in expression during vernalisation. It is the only gene that fully meets all four criteria and thus serves as the primary candidate for storing vernalisation memory in temperate cereals. This is reminiscent of the conclusive experiments in Arabidopsis (Berry et al., 2015), which demonstrated that the epigenetic memory of winter is stored at the *FLC* locus through localised and heritable changes in H3K27me3. Such an experiment in cereals would be able to directly test whether vernalisation memory is confined to a single locus like *VRN1*, or distributed among multiple sites. However, it cannot be excluded at this stage that other genes such as *VRN3* and *ODDSOC2* may also contribute to vernalisation memory, potentially indicating a broader network of epigenetically regulated genes. Further research will be required to determine whether memory is restricted to a single locus or distributed among multiple sites.

## Epigenetic gene activation of *VRN1*

**Figure 3:**
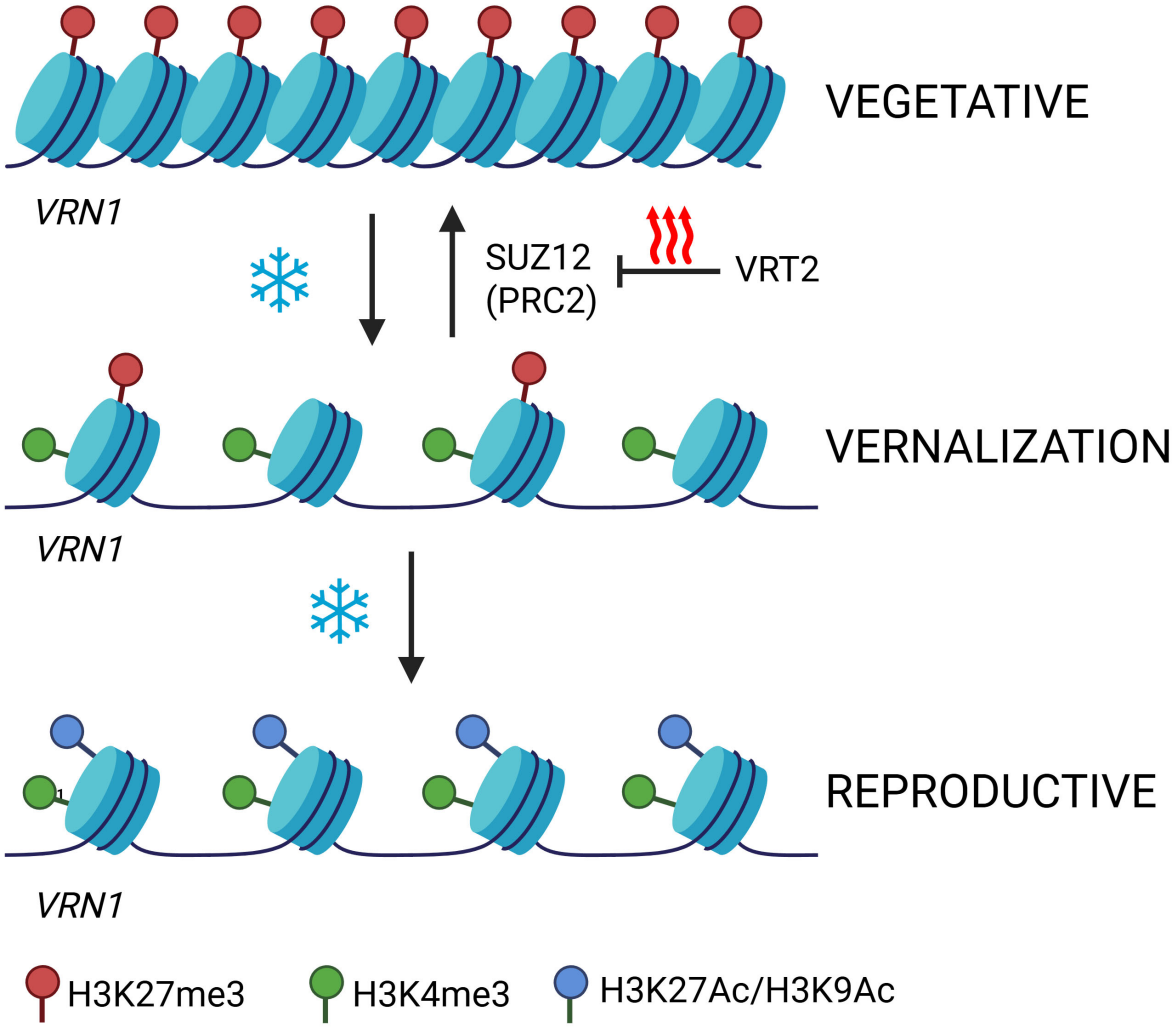
Epigenetic activation *VRN1* locus by vernalisation. The figure depicts the epigenetic regulation of the *VRN1* locus during vernalisation in temperate cereals as a continuous process. Before cold exposure, *VRN1* chromatin is compact and enriched in the repressive mark H3K27me3 (red), keeping the gene silent. During prolonged cold, H3K27me3 levels gradually decrease while activating marks such as H3K4me3 (green) and H3K27Ac or H3K9Ac (blue) accumulate, leading to chromatin opening and VRN1 activation. After vernalisation, in warm conditions, *VRN1* remains actively transcribed with sustained H3K4me3, H3K27Ac, H3K9Ac, and H3K36me3 marks, which stabilise expression and prevent re-silencing. The transcription factor VRT2 regulates the PRC2 component SUZ12 to block re-deposition of H3K27me3, ensuring the vernalised, active state is maintained. Colours: red, H3K27me3; green, H3K4me3; blue, acetylation. Created in BioRender. Daneels, T. (2025) https://BioRender.com/v14g358.

In temperate cereals, the epigenetic regulation of vernalisation memory centres on the activation of the flowering promoter *VRN1*, in contrast to the silencing-based mechanism observed in Arabidopsis. Upon exposure to prolonged cold, H3K27me3 levels at the *VRN1* locus gradually decrease, relieving repression and allowing the gene to be activated (**Figure 3**) (Huan et al., 2018; Oliver et al., 2009). This loss of the repressive mark is an early and necessary step in the transition from a silent to an active chromatin state. As H3K27me3 is removed, activating histone modifications, most notably H3K4me3, accumulate at the *VRN1* promoter and 5′ region (Liu Y. et al., 2024; Oliver et al., 2009). The presence of both H3K27me3 and H3K4me3 at the *VRN1* locus may represent a poised or bivalent chromatin state, keeping the gene responsive to environmental cues and primed for full activation (Azuara et al., 2006; Bernstein et al., 2006).

As vernalisation progresses, additional activating marks such as H3K27ac and H3K9ac (acetylation of histone H3 at lysine 9) are enriched at the *VRN1* promoter, further loosening chromatin structure and facilitating transcriptional activation (Liu Y. et al., 2024; Wang et al., 2009). After transcription is initiated, H3K36me3 is deposited across the gene body, supporting transcriptional fidelity and preventing the re-establishment of repressive marks (Finogenova et al., 2020; Yuan et al., 2011). This sequence, initial loss of H3K27me3, accumulation of H3K4me3 and histone acetylation, followed by H3K36me3 deposition, suggests a stepwise transition from a repressed to an active and stably expressed state. However, the precise chromatin mark or combination of marks responsible for storing the epigenetic memory of vernalisation in cereals remains to be fully determined.

However, the crosstalk and feedback between these histone marks add a layer of complexity, as the presence or removal of one modification can influence the recruitment or exclusion of enzymes responsible for other marks (Janssen and Lorincz, 2022). For example, the removal of H3K27me3 may facilitate the recruitment of H3K4 methyltransferases (Schmitges et al., 2011), while the establishment of H3K36me3 can inhibit the re-deposition of H3K27me3 (Yuan et al., 2011), reinforcing the active state. These dynamic and interdependent relationships underscore that epigenetic regulation is rarely dictated by a single mark, but rather by coordinated modifications that collectively determine chromatin state and gene expression. Recent work in Arabidopsis further illustrates that not only the interplay but also the timing of these histone modifications is critical; dynamic changes in chromatin marks can serve as molecular timers during developmental transitions, ensuring that gene activation is precisely integrated with developmental cues (Huang et al., 2021).

A defining feature of vernalisation memory is its persistence: once established, the vernalised state is generally maintained even if plants are subsequently exposed to high temperatures. Recent research in Brachypodium has shed light on the molecular mechanisms that safeguard this memory from reversal. Kennedy et al. (2024) demonstrated that the MADS-box transcription factor VRT2 plays a crucial role in this process by transcriptionally regulating the PRC2 component SUZ12. By modulating SUZ12 expression, VRT2 may influence the deposition of H3K27me3 at key flowering loci, thereby preventing the re-establishment of a repressive chromatin state and ensuring the irreversibility of vernalisation memory even under fluctuating temperatures. This finding highlights VRT2 as a potential guardian of vernalisation memory, acting to stabilise the epigenetic state acquired during cold exposure (Kennedy et al., 2024). Future studies will be important to further elucidate the precise molecular mechanisms underlying this regulation and to explore how these pathways can be manipulated to improve crop adaptation and flowering time control.

## Conclusion and future perspectives

It is still unclear whether vernalisation memory in temperate cereals is stored at a single gene locus or spread across multiple genes. However, the strongest evidence points to a central role for *VRN1* and the dynamic changes in its chromatin state. The balance between repressive and activating histone marks at *VRN1* appears to be critical, although the exact order and mechanisms remain to be fully defined. Future research should focus on understanding how these epigenetic modifications are established and maintained, and whether other genes also contribute to vernalisation memory. Such discoveries will be vital for deepening our knowledge of plant adaptation to seasonal cues and for improving crop resilience in a changing climate.
